# Development of a Forced Degradation Profile of Alosetron by Single Mode Reversed-Phase HPLC, LC-MS, and its Validation

**DOI:** 10.3797/scipharm.1411-07

**Published:** 2014-12-29

**Authors:** Yamjala Karthik, Basuvan Babu, Subramania Nainar Meyyanathan

**Affiliations:** Department of Pharmaceutical Analysis, JSS College of Pharmacy (A Constituent College of JSS University, Mysore), Udhagamandalam, The Nilgiris, Tamilnadu, 643001, India

**Keywords:** Alosetron, Degradation, ICH, Stress studies, Validation

## Abstract

Determination of alosetron in the presence of its degradation products was studied and validated by a novel HPLC method. The separation of the drug and its degradation products was achieved with the Jones Chromatography C_18_ analytical column (150 mm x 4.6 mm; 3 µm) with a stationary phase in isocratic elution mode. The mobile phase used was 0.01 M ammonium acetate, pH-adjusted to 3.5 with glacial acetic acid and acetonitrile in the ratio of 75:25 (V/V) at a flow rate of 1 ml/min and UV detection was carried out at 217 nm. Further, the drug was subjected to stress studies for acidic, basic, neutral, oxidative, and thermal degradations as per ICH guidelines and the drug was found to be labile in base hydrolysis and oxidation, while stable in acid, neutral, thermal, and photolytic degradation conditions. An MS study has been performed on the major degradation products to predict the degradation pathway of alosetron. The method provided linear responses over the concentration range of 100–1500 ng/ml and regression analysis showed a correlation coefficient value (r^2^) of 0.994. The LOD and LOQ were found to be 1 ng/ml and 3 ng/ml, respectively. The developed LC method was validated as per ICH guidelines with respect to accuracy, selectivity, precision, linearity, and robustness.

## Introduction

**Fig.1:**
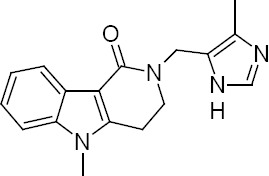
Chemical structure of alosetron

Alosetron (Figure 1) is a 5-HT_3_ antagonist used in the prophylaxis treatment for women with predominant irritable bowel syndrome (IBS) [1, 2]. IBS is a gastrointestinal disorder characterized by abdominal pain, distressed bowel function, and abdominal distension. IBS is diagnosed more frequently in women and typically in patients < 50 years of age [3]. Alosetron was included in the United States Pharmacopeia (USP) prioritized list of chemical medicine monographs in 2013 [4]. Review of the literature revealed that only a few studies have been performed for the estimation of alosetron. Thomas L. Lloyd *et al*. have reported the estimation of alosetron in human plasma or serum by high-performance liquid chromatography employing robotic sample preparation, cyanopropyl stationary phase, and fluorescence detection [5]. Wring SA *et al*. have reported the radioimmunoassay method for the estimation of alosetron in human urine and saliva [6]. Karthik *et al*. have proposed an HPLC method for the estimation of alosetron in bulk drug employing a UV detector for the first time [7].

The ICH guidelines [8] suggest that stress degradation studies on a drug is carried out to establish its inherent stability characteristics not only for identification of stress degradation products, but also for understanding the stability of a drug molecule. So it is of immense importance to know the complete degradation profile of alosetron. To date, there is no reported stability-indicating assay method for the estimation of alosetron by reversed-phase high-performance liquid chromatography, hence we determined to develop a validated stability-indicating analytical assay method. The present study describes: i. Forced degradation studies on alosetron (acid, base, neutral, thermal hydrolysis, photolytic, and oxidative degradation), ii. Optimization of the method to separate degradation products from the drug, iii. Validation of the developed method as per international guidelines, and iv. An attempt has been made to predict possible degradation pathways by employing LC-MS.

## Experimental

### Materials

Alosetron HCl (>99.5%) was provided by Orchid Laboratories (Chennai). Acetonitrile for HPLC was procured from Merck Limited (Mumbai, India). Glacial acetic acid and ammonium acetate of HPLC and LC-MS grades were purchased from S.D Fine Chemicals (Mumbai, India). Water of HPLC grade, obtained from the Milli-Q RO system, was used in the preparation of the buffer and sample solutions.

### Apparatus

The HPLC system was from Shimadzu (Kyoto, Japan) with an LC-10 AT-VP solvent delivery system, SPD M10 A UV detector, LC-2010 A HT autosampler with a loop volume of 100 µl, and a Class VP data station. LC–MS studies were performed by the Shimadzu LC-2020 quadrapole mass spectrometer with an ESI source in positive mode equipped with LC-10AD gradient pumps, a DGU-14AM degasser, SCL-10A system controller, CTO-10A column oven, diode array detector (SPD-M10A), and an autoinjector (SIL-10AD) (all from Shimadzu, Kyoto, Japan). The data was acquired and processed using LC lab solutions software. The stationary phase used for separation was the Jones Chromatography C_18_ analytical column. The absorption maximum of alosetron was determined by using a Shimadzu 1700 spectrophotometer. An ultraviolet lamp at 245 nm and 365 nm for the stress degradation was employed.

### Stress Degradation Studies

The stress degradation study on alosetron was performed as per ICH guideline Q1A (R2) [8]. For each forced degradation condition, the sample was dissolved in 25 ml of water and degradation samples were prepared with acid, alkali, hydrogen peroxide, and water to get a concentration of 1000 µg/ml and it was subjected to different stress conditions by refluxing for 24 hr with each 25 ml of 0.1 N HCl, 0.1 N NaOH, 3% H_2_O_2_, and water at 60ºC. The powdered drug was placed in an oven at 80ºC in solid form and under UV light at 245 nm and 365 nm for 24 hr. After particular time intervals, the samples were withdrawn and stored at 4°C.

### Preparation of Sample Solutions

Samples of acid and base hydrolysis were neutralized by 0.1 N NaOH and 0.1 N HCl, respectively. The solutions of acid, base, thermal, photolytic, neutral, and peroxide degradation were diluted with mobile phase to obtain a concentration of 100 µg/ml before injecting into the system.

### Validation

The proposed method was validated as per ICH guidelines [9]. Standard solution of alosetron (1 mg/ml) was diluted to obtain solutions in the concentration range of 100–1500 ng/ml to ascertain the linearity and range. The solutions were injected in triplicate of about 20 µl. The calibration curve was plotted against the peak areas and corresponding concentrations. Intraday and interday precisions were established by analyzing 250, 500, and 750 ng/ml concentrations in triplicate on the same day and on three consecutive days, respectively. Accuracy was determined in terms of recovery by analyzing a known concentration of the drug, viz. 250, 500, and 750 ng/ml spiked with the degradation sample in triplicate and then calculating the percent recovery. The S/N ratio method was used to determine the detection and quantification limits. The robustness of the method was determined by minor deliberate changes in the chromatographic conditions.

## Results

### Method Development

Various solvent systems such as acetonitrile-water, methanol-water, methanol-buffer, and buffer-acetonitrile mixtures in different ratios, having different pH ranges (2.5–8) and flow rates (0.8–1.2 ml/min), were evaluated. The best separation and resolution between the drug and degradation products were achieved by using 0.01 M of ammonium acetate buffer (pH-adjusted to 3.0 using glacial acetic acid) and acetonitrile in the ratio of 75:25 V/V as the mobile phase and the Jones Chromatography C_18_ (150 mm x 4.6 mm; 3 µm particle size) column as the stationary phase. The analyses were carried out in isocratic elution mode using a flow rate of 1.0 ml/min, injection volume of 20 µl at room temperature, and the detection of an analyte was recorded at 217 nm. The mobile phase solvents were filtered through a 0.22 µm polytetrafluoroethylene membrane filter before injecting into the HPLC system and the chromatograms were recorded using Class VP software.

### Validation

The various chromatographic parameters such as retention time (R_t_), relative retention time (RRT), resolution factor (R_s_), and asymmetry factor (A_s_) are listed in Table 1. The developed stability-indicating method was validated as per ICH guidelines in terms of linearity, precision, accuracy, specificity, detection, and quantification limits. The detector response for the drug was found to be linear over the concentration range of 100-1500 ng/ml (r^2^=0.994; n=6). Accuracy was calculated in terms of percent recovery upon spiking a combination of stressed samples with the three known concentrations of the real samples, viz., 250, 500, and 750 ng/ml and the results were found to be between 99.46%-100.17% (Table 2). Precision results (intraday and interday) are shown in Table 3. The % RSD values for the intra- and interprecision studies were found to be less than 1.9 and 1.4, respectively, which confirmed that the developed method was adequately precise. Detection and quantification limits were found to be 1 ng/ml and 3 ng/ml, respectively, which indicate the method is more sensitive than the reported methods. The method was successfully applied to real samples and results are shown in Table 4. Minor changes in the optimized chromatographic conditions did not influence the retention time of the drug, indicating the robustness of the method. System suitability parameters such as theoretical plates (N) and tailing factor (T_f_) for the drug were found to be 5301 per meter and 1.08, respectively.

**Tab. 1:**
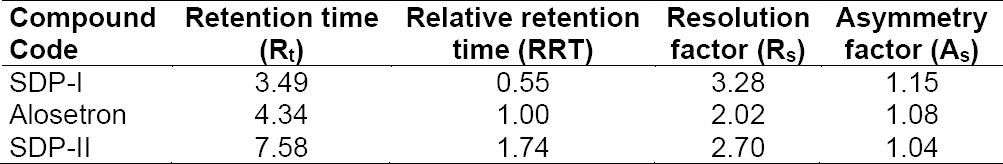
Chromatographic data

**Tab. 2:**
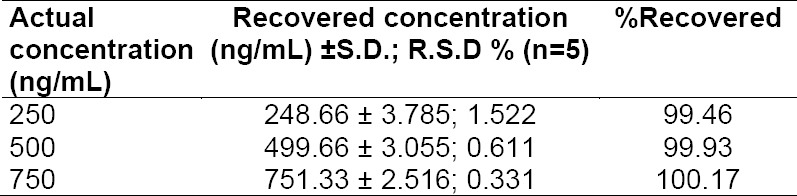
Recovery studies

**Tab. 3:**
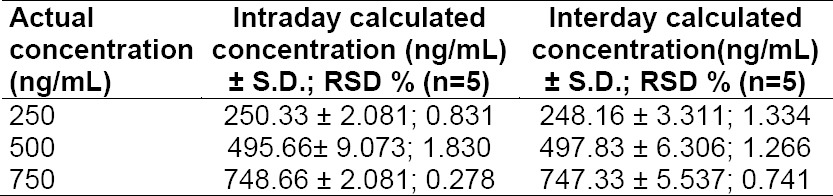
Precision studies

**Tab. 4:**
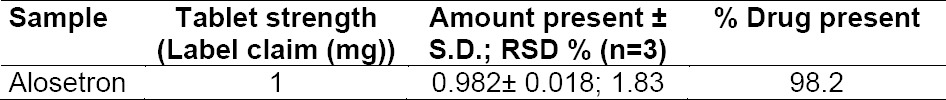
Assay of formulation

## Discussion

### Stress Degradation

**Fig. 2:**
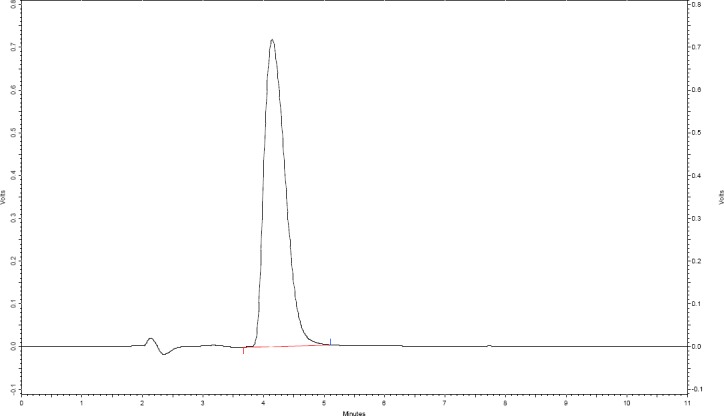
Standard HPLC-UV chromatogram of alosetron

**Fig. 3:**
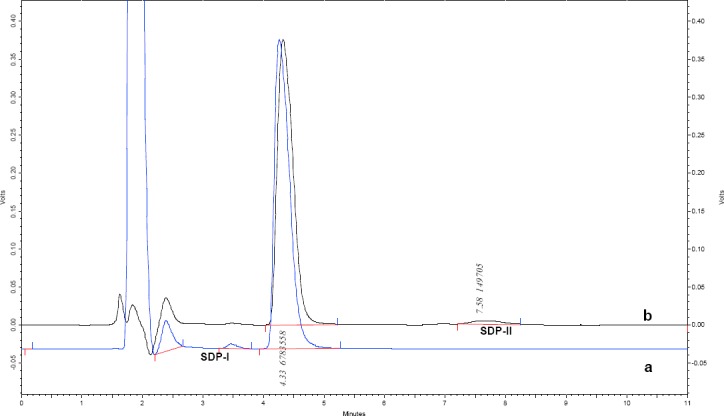
Overlay chromatograms of alosetron showing (a) oxidative degradation and (b) base hydrolysis

**Fig. 4:**
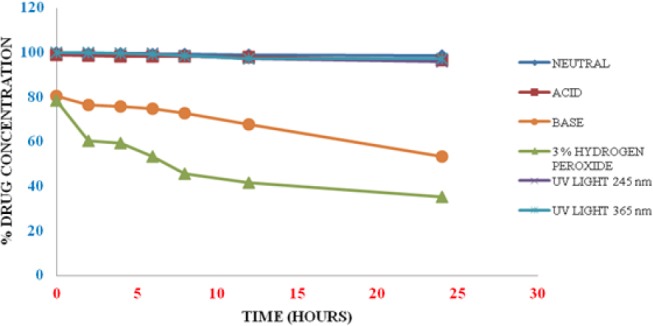
Degradation behavior of alosetron under various stress conditions

The degradation of alosetron under various forced degradation conditions like acid, base, neutral, oxidation, thermal, and photolytic under UV light were examined by liquid chromatography and the chromatograms were recorded. The typical HPLC chromatogram of the drug and chromatogram showing separation degradation products are depicted in Figures 2 and 3, respectively. The degradation behavior of alosetron under various stress conditions is shown in Figure 4.

### Acid, Base, and Neutral Hydrolysis

In base hydrolysis, degradation more than 40% was observed with one degradation product (SDP-II) after 24 hr. However, no degradation peak was observed in the case of acid and neutral hydrolysis.

### Oxidative Degradation

The drug was tested under 3% hydrogen peroxide for 24 hr and the degradation was greater than 60%. One degradation product was observed (SDP-I) under oxidative stress.

### Thermal Degradation

Alosetron was kept in solid form at 80°C for 24 hr. No significant degradation was observed. Hence, the drug was found to be stable under thermal degradation.

### Photolytic Degradation

The solution of alosetron 100 µg/ml was irradiated under UV light at 245 nm and 365 nm for 24 hr and the chromatograms were recorded. No major degradation was observed.

### MS Studies

**Fig. 5:**
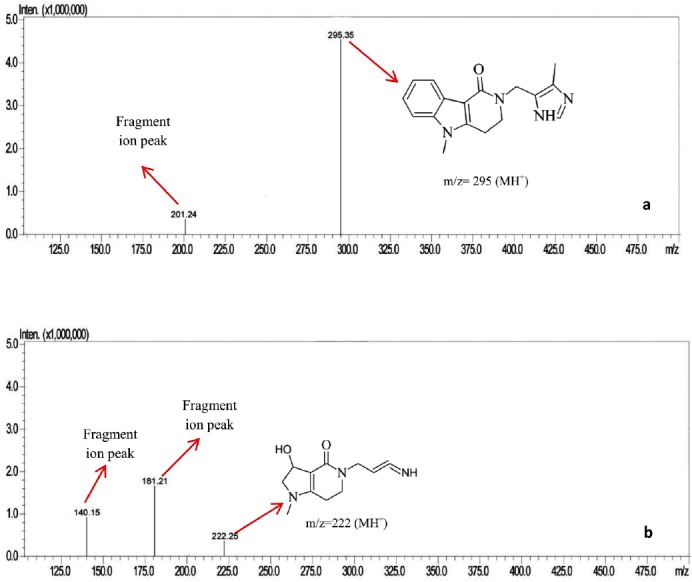
Mass spectra of alosetron and (a) SDP-II (b)

**Fig. 6:**
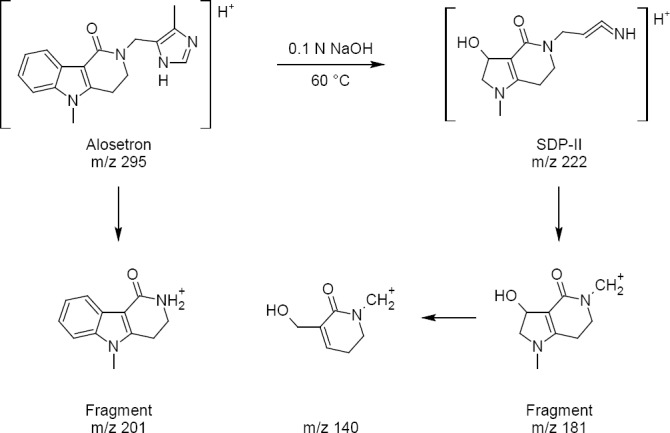
Predicted degradation pathway of alosetron in alkaline medium

SDP-II was the major degradation product observed in alkaline hydrolysis, so we determined to conduct an MS study to identify the molecular ion peak of the degradation product. For that we have isolated SDP-II by employing a semi-preparative HPLC column. The samples (10 µL) were injected directly into the source by a flow injection method using water and acetonitrile in the ratio 20: 80 v/v as the mobile phase at a flow rate of 0.5 mL/min. Ultra high purity nitrogen was used as the drying gas. The typical ion source conditions were: nebulizer gas, 60 psi; dry temperature, 350°C; dry gas, 5.0 mL/min; capillary voltage, 5kV; vaporizer temperature, 400°C; and dwell time, 200 ms. In ESI positive ion mode, the mass spectrum of the drug and SDP-II has shown molecular ion peaks at m/z values of 295 and 222, respectively (Figures 5a and 5b). The mass spectra also showed a few fragment ions at various m/z values i.e., 201 for the drug, and 140 and 181 for SDP-II. Based on the molecular ion peak of the SDP-II, the possible degradation pathway of alosetron was predicted which is shown in Figure 6.

## Conclusion

A novel stability-indicating liquid chromatography method has been developed using a UV detector. An attempt was made to explore the degradation behavior of alosetron by exposing it to ICH-defined stress degradation conditions. The developed method was able to separate alosetron from its degradation products (SDP-I and SDP-II) with a minimal resolution of 2.02. The drug was found to be stable under acid, neutral, hydrolysis, thermal degradation, and photolysis conditions. One degradation product each was formed under oxidative degradation (SDP-I) and base hydrolysis (SDP-II). The major degradation product (SDP-II) may be 3-hydroxy-5-(3-iminoprop-2-en-1-yl)-1-methyl-1,2,3,5,6,7-hexahydro-4*H*-pyrrolo[3,2-*c*]pyridin-4-one. Thus, the developed method can also be used for the identification of stress degradation products along with routine quality control analysis.
